# Concrete incorporating supplementary cementitious materials: Temporal evolution of compressive strength and environmental life cycle assessment

**DOI:** 10.1016/j.heliyon.2024.e25056

**Published:** 2024-01-22

**Authors:** Noor Yaseen, Stefany Alcivar-Bastidas, Muhammad Irfan-ul-Hassan, Daniel M. Petroche, Asad Ullah Qazi, Angel D. Ramirez

**Affiliations:** a Department of Civil Engineering, University of Engineering and Technology, Lahore, Pakistan; b Directorate General Small Dams, Irrigation Department, Khyber, Pakhtunkhwa, Pakistan; c Facultad de Ingeniería, Universidad Católica de Santiago de Guayaquil, Av. Carlos Julio Arosemena Km 1 1/2, P.O. Box 10369, Guayaquil, Ecuador; d Facultad de Ingeniería en Mecánica y Ciencias de la Producción, Escuela Superior Politécnica del Litoral, ESPOL, Campus Gustavo Galindo, Km. 30.5 Vía Perimetral, P.O. Box 09-01-5863, Guayaquil, Ecuador

**Keywords:** Compressive strength, *fib* model Code 2010, Fly ash, Silica fume, Strength evolution model, LCA

## Abstract

The use of Supplementary Cementitious Materials (SCMs) or industrial wastes as a partial replacement for cement in the production of concrete is an urgent need in the construction industry due to cement's growing environmental challenges and rising cost. In respect of this, we conducted research work on proportioning binary concrete mixes. Fly ash (FA) replaced 10 %, 20 %, and 30 % of the cement, while silica fume (SF) replaced 5 %, 10 %, and 15 % of the cement. A control concrete mix was also developed with 100 % cement and no SCM. The results showed no increase in compressive strength for FA concrete compared to control at the early age of 3–28 days, but a maximum increase in compressive strength of 4 % was discovered at a later age of 56 days for concrete with 20 % FA. For 5 % SF concrete, a considerable strength increase of 15 % was seen at the early age of 3 days. Like with FA concrete, 2 % improvement in strength was recorded at the later age of 56 days for 10 % SF concrete. This study further focused on the concrete's temporal evolution of compressive strength by developing a strength evolution model (SEM) using nonlinear regression analysis at a 95 % confidence level. Pearson correlation coefficient was used to determine the correlation between the model values and the experimental results. For comparison, the fib Model Code 2010 was applied to the experimental data, and a good agreement was observed among the proposed model, the fib Model values, and the experimental results. The proposed model can be expanded to address further regression-related problems. Finally, environmental life cycle assessment revealed that utilizing 10 %, 20 %, and 30 % of FA lowered Global Warming Potential (GWP) by 9 %, 19 %, and 29 %, respectively. Likewise, using 5 %, 10 %, and 15 % of SF reduced the GWP by 5 %, 9 %, and 14 %.

## 1 Introduction

Cement is one of the essential components of concrete production. Although it is manufactured commercially on a large scale, its production leads to resource depletion and leaves a substantial carbon footprint. In fact, 5–8 % of global CO_2_ emissions are attributed to cement production [[1], [2], [3], [4], [5], [6], [7]]. The escalating demand for sustainable development has prompted researchers to develop more efficient clinker, cement, and concrete production processes, explore composite cement containing supplementary cementitious materials (SCMs), and innovate in concrete production by integrating biomaterials or industrial wastes as a partial cement replacement [[8], [9], [10], [11], [12], [13], [14], [15], [16]]. The use of fly ash and silica fume as a partial cement replacement holds significant potential for sustainable construction by minimizing greenhouse gases, achieving CO_2_ reductions (Global Warming Potential GWP) between 10 and 25 % depending on the fly ash and silica fume content [[17], [18], [19], [20]], and reducing industrial waste products and the associated waste disposal issues [21].

In light of the rationale above, numerous studies have been conducted and published in the scientific literature on concrete incorporating fly ash and silica fume, either individually or in combination. Golewski, for example, demonstrated that the compressive strength and water absorption of concrete were greatly influenced by the percentage of fly ash used [22]. This researcher found that adding 20 % fly ash increased the compressive strength and water absorption of concrete; however, both compressive strength and water absorption decreased with a further increase to 30 % fly ash. This indicates that while the increasing content of fly ash beyond an optimum percentage may decrease the compressive strength, it can enhance the microstructure of the composite by filling the pores [23].

A combined effect of fly ash and silica fume can also be achieved with proper mix proportions. For instance, a concrete mix with 10 % fly ash and 10 % silica fume resulted in a compressive strength of 48 MPa at a 7-day curing age and above 63 MPa at a 28-day curing age [24]. This is possible by reducing the water-binder ratio (w/b) to get the desired strength and using super-plasticizers to maintain the required workability [[25], [26], [27]]. In this sense, Wu et al. [26] examined the mechanical properties of coral aggregate concrete incorporating fly ash and silica fume. They found that fly ash had an adverse influence on strength at an early age, but the strength was significant at later ages, whereas the strength at all ages increased with the addition of silica fume. Nassif et al. [28] investigated the influence of fly ash and silica fume on the elastic modulus of high-performance concrete. They observed that mixes with 10 % fly ash and no silica fume did not significantly affect strength; however, the elastic modulus increased at all ages. In contrast, mixes with 10 % silica fume and no-fly ash increased strength at all ages, but the modulus was increased only at early ages. The increase in modulus for concretes with 10 % silica fume and no-fly ash at early ages is attributed to the higher pozzolanic activity of silica fume.

While adding fly ash to concrete mixes as a partial replacement for cement increases the later-age strength of the concrete, it may reduce the early-age strength, a limitation that can be mitigated by utilizing nanomaterials like nanosilica [29]. In a study by Golewski, it was found that concrete with quaternary compositions (80 % cement, 5 % fly ash, 10 % silica fume, and 5 % nanosilica) demonstrated higher strength parameters and enhanced microstructure [[30], [31], [32]]. Considering these research findings and others reported in the literature, it can be inferred that using SCMs like fly ash and silica fume can significantly reduce cement consumption without compromising the performance and durability of concrete structures.

Predicting concrete compressive strength is of utmost importance in design calculations of concrete structures, specifically at a curing age of 28 days, as demonstrated by Ausweger et al. [33]. These researchers also improved the formulae of the *fib* Model Code 2010 [34], the correlation model, relating the 28-day compressive strength and the stiffness, and the evolution formula, which links the early-age evolution of compressive strength and the stiffness up to the 28-day age of the concrete [33]. The wealth of studies and literature on predicting the compressive strength of concrete with fly ash and silica fume using different statistical methods reflects the growing interest in understanding and optimizing the performance of concrete mixtures containing these additives [[35], [36], [37], [38], [39], [40]]. Researchers have conducted numerous experiments and analyses to develop empirical models that can predict the compressive strength of concrete incorporating fly ash and silica fume. For example, Pala et al. [41] used the neural network (NN) method to predict the compressive strength of concrete with fly ash and silica fume. Their findings indicated that fly ash contributed minimally to the compressive strength at an early age but significantly increased it at a later age of curing. Additionally, it was concluded that adding a small amount of silica fume had an insignificant impact on compressive strength.

Recent literature has demonstrated the significant role of statistical modeling in determining the relationship between factors like fly ash content, silica fume content, and water-cement ratio and their influence on the compressive strength of concrete. However, methods such as neural networks and machine learning may involve time-intensive and complex calculations when predicting concrete properties, as Behnood and Golafshani [40] did. Regression analysis, on the other hand, as a simple statistical technique, helps to establish mathematical equations or models that can predict the mechanical properties of concrete based on input variables. Several researchers have reported that, besides offering better accuracy, the main advantage of regression analysis is its ease of application [42]. These regression models play a crucial role in designing and optimizing concrete mixtures by providing a means to estimate the expected mechanical properties before conducting time and resource-intensive laboratory tests. In this regard, Gupta et al. [43] explored the incorporation of varying percentages of fly ash in eighteen mixes and developed predictive models for compressive strength using linear regression analysis, artificial neural network, and leave-one-out validation methods. Similarly, Abd Elaty conducted a regression analysis on experimental compressive strength results and developed a logarithmic model for predicting compressive strength at any curing age of concrete [44]. Researchers have widely used the Pearson correlation coefficient to determine correlations between different properties of cementitious materials. For instance, Lin et al. utilized Pearson correlation analysis to assess the effects of silica fume and steel fiber on the engineering parameters of fiber cementitious materials [45]. Likewise, other studies have evaluated models using the Pearson correlation coefficient [[46], [47], [48]]. This research employed regression and correlation analyses, utilizing the 95 % confidence interval and the Pearson correlation coefficient to evaluate model accuracy and investigate the association between the model and experimental results.

Despite significant research on fly ash and silica fume in concrete, there remains a need for deeper exploration into their specific impact on concrete strength. Furthermore, the temporal evolution of compressive strength in concrete with these supplementary cementitious materials (SCMs) and the development of corresponding regression models predicting compressive strength have received relatively less attention. Additionally, the incorporation of fly ash and silica fume in concrete could provide environmental benefits that need to be quantified through life cycle assessment (LCA). Therefore, this work aims to determine the individual effect of fly ash and silica fume on concrete strength, develop the best-fit model with minimum input parameters, and assess the global warming potential of employing these SCMs in concrete.

### 1.1 Objectives and tasks

**Fig. 1:**
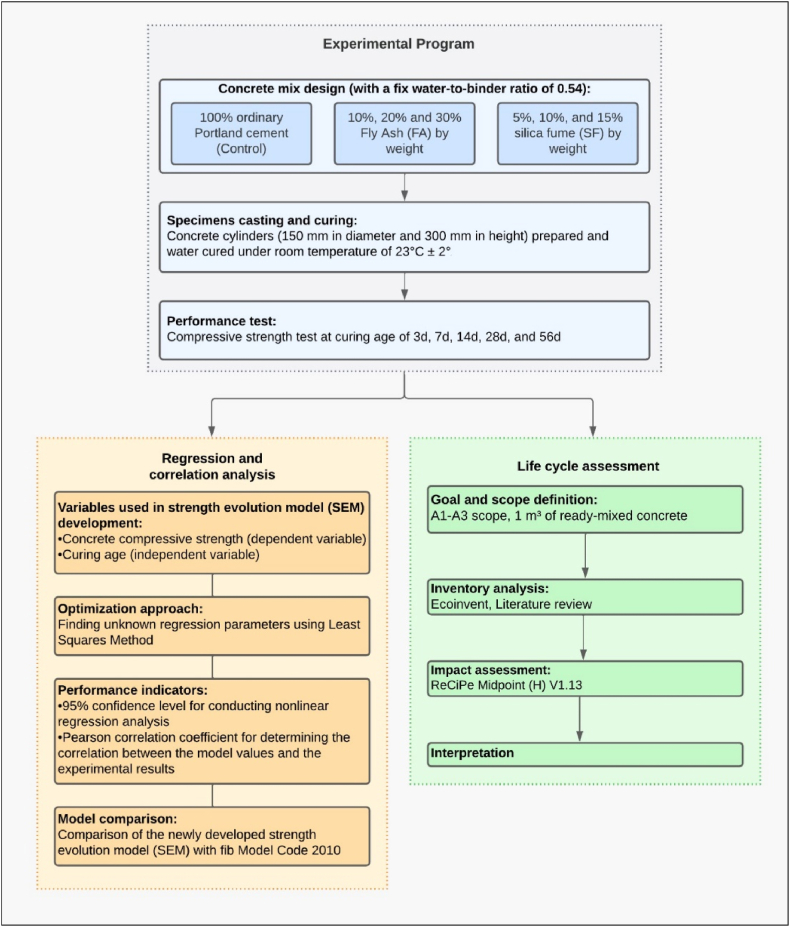
Block diagram of proposed research methodology.

This research focused on the temporal evolution of concrete compressive strength (*f*_*c*_) using binary blends of fly ash (FA) and silica fume (SF) as partial cement replacements. The experimental program of this study involved concrete with and without supplementary cementitious materials (SCMs) with a fixed water-binder ratio (w/b) of 0.54. The mix design included seven mixes: a control mix (100 % cement), 10 % FA, 20 % FA, 30 % SF, 05%SF, 10 % SF and 15 % SF. The relative mass ratio of the concrete mixes was kept constant, with the only change in the proportions of pozzolans. The prepared specimens were tested for compressive strength at different ages: 3, 7, 14, 28, and 56 days. From the experimental results, the aim was to develop and explore a new strength evolution model for concrete with SCMs using regression analysis. Based on the experimental findings, a nonlinear regression equation that correlated *f*_*c*_ with the concrete age, *t,* was developed. In addition, the *fib* Model Code 2010 [34] was applied to the experimental data in order to compare the results of the newly developed model. Finally, the influence of FA and SF on the environmental profile of concrete was calculated using life cycle assessment (LCA). A block diagram (an overview) showcasing the different aspects of the study in relation to its objectives and tasks is show in Fig. 1.

## 2 Materials and methods

### 2.1 Materials

**Fig. 2:**
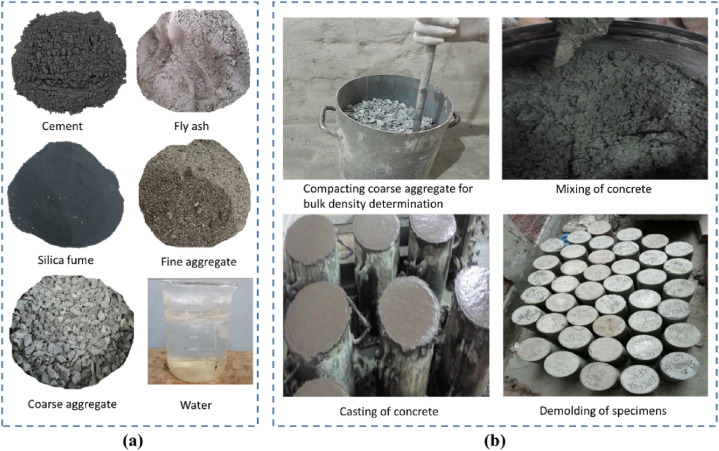
Stages of concrete samples preparation: Appearance of materials used in concrete mix design (**a**), and processes involved in preparation of concrete specimens **(b)**.

Fig. 2 shows the stages of concrete production including visual appearance of various materials used in mix design (Fig. 2a) and the processes involved in the concrete samples preparation (Fig. 2b). Ordinary Portland Cement (OPC) was used as a basic cementing material for the mix design of concrete, according to ASTM C150 standards [49]. The cement used was grey, with a specific gravity of 3.14. Fly ash (FA) was categorized as class F according to ASTM C618 [50] and was sourced from a coal-burning power plant through a commercial supplier in Lahore, Pakistan. The FA was brown with a specific gravity of 2.29. Furthermore, the grey-colored silica fume (SF) was obtained from a local supplier in Karachi and met the specifications outlined in ASTM C1240-15 [51]. Silica fume consisted of spherical particles with an average diameter of 150 nm. Well-graded Lawrencepur sand and Sargodha crush, procured from a local market, were used as fine aggregate and coarse aggregate in preparing concrete specimens. The maximum allowable grain sizes for fine and coarse aggregates were 4.75 mm and 13 mm, respectively. Saturated surface dry condition (SSD) was maintained for both fine and coarse aggregates. Table 1 shows the physical properties of fine and coarse aggregates determined using ASTM methods. Potable water was used for the mixing of materials and the curing of concrete specimens.

**Table 1:** Physical properties of fine aggregate and coarse aggregate.

Property	Fine aggregate	Coarse aggregate	Test method	Reference
Compacted bulk density (kg/m^3^)	1720	1456	ASTM C29	[52]
Bulk specific gravity (SSD)	2.63	2.57	ASTM C128 & ASTM C127	[53,54]
Water absorption (%)	1.75	1.31	ASTM C128 & ASTM C127	[53,54]
Fineness modulus	2.63	–	ASTM C136	[55]

### 2.2 Concrete mixing and casting

In this phase, we aimed to target the conventional concrete of 28 MPa, widely used in residential and frame-structure constructions worldwide. For that purpose, we conducted the mix design for the control mix (100 % cement and no SCM) and for mixes containing SCMs according to ACI 211.1–91 [56], as detailed in Table 2. The water-binder ratio of 0.54 was maintained throughout all mix proportions, the relative mass ratio of sand, aggregates, and binder (cement + SCMs) also remained the same for all mix proportions. Different proportions of FA and SF were used to substitute the cement with an equivalent water-to-binder ratio of 0.54. The replacements included 10 %, 20 %, and 30 % by weight of binder for FA and 5 %, 10 %, and 15 % by weight of the binder for SF. These mixes were represented as FA10, FA20, FA30, SF05, SF10, and SF15.

**Table 2:** Mix design for 1 m^3^ of concrete.

Mix	w/binder	Mix proportions of concrete (kg/m^3^)					
		OPC	FA	SF	Fine aggregate	Coarse aggregate	Water
Control	0.54	507	0	0	688	1116	274
FA10	0.54	456	51	0	688	1116	274
FA20	0.54	406	101	0	688	1116	274
FA30	0.54	355	152	0	688	1116	274
SF05	0.54	482	0	25	688	1116	274
SF10	0.54	457	0	51	688	1116	274
SF15	0.54	431	0	76	688	1116	274

A rotary mixer was used for the mixing process. Initially, the cement, sand, and coarse aggregate were dry mixed; then, water at a water-binder of 0.54 was added and mixed for 3 min, followed by adding the corresponding SCM for 5 min. The resulting mixture was poured into cylinders with a diameter of 150 mm and a height of 300 mm in three layers and compacted using a vibrating table. After 24 h of casting, the specimens were removed from the molds. The curing of the specimens was carried out in potable water at an ambient temperature of 23 °C ± 2 °C throughout the entire curing period until tested under compression. The same procedure was replicated for the preparation of control specimens. All specimens were tested at ages 3, 7, 14, 28, and 56 days to determine their compressive strengths.

### 2.3 Compressive strength test

**Fig. 3:**
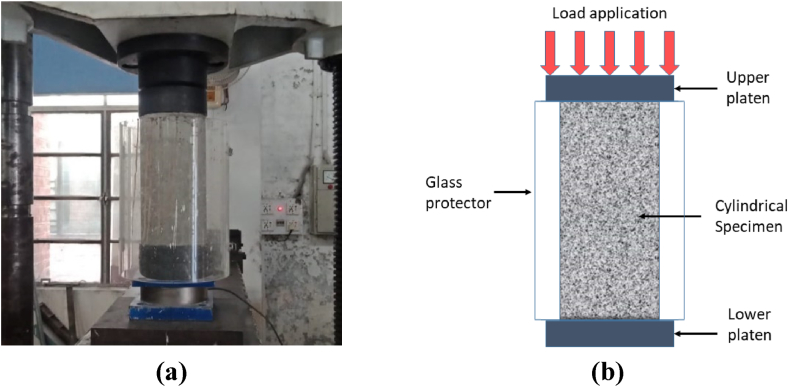
Compressive strength test setup: Concrete cylinder and compression testing machine **(a)**, and schematic of compression test setup **(b)**.

Concrete cylinders were subjected to destructive compression tests in accordance with ASTM C39/C39M − 21 [57], using a displacement-controlled universal testing machine (UTM), as depicted in Fig. 3a and a schematic of compression test setup as shown in Fig. 3 b. Each mix underwent testing using three specimens under compression to obtain the average compressive strength.

### 2.4 Regression and correlation analysis

Verifying experimental data is crucial for ensuring its reliability before performing regression analysis. In this study, an analytical model based on multiscale-micromechanics provided by Königsberger et al. [58] was applied to the control specimens to validate the experimental data related to concrete compressive strength (Appendix A). Subsequently, after determining and evaluating the experimental values for both control concrete and concrete with SCMs, a strength evolution model (SEM) was developed to estimate the compressive strength of concrete with and without SCMs. Based on the experimental compressive strength results, the best-fit nonlinear regression equations were developed with a 95 % confidence level for all concrete mixtures, and the results were graphically plotted for visual inspection. The regression analysis in this study was conducted using the software 'Minitab-18'. Furthermore, Pearson correlation analysis, a straightforward statistical method, was employed as part of the research methodology to assess the correlation between variables. The Pearson correlation coefficient (represented as *r*) is a pure number used for determining the linear relationship between two variables, with values ranging from −1 to +1 [59]. It is important to note that a positive correlation is indicated by *r* > 0, where +1 signifies a perfect positive linear relationship, and conversely, *r* < 0 denotes a negative correlation, with −1 indicating a perfect negative linear relationship [[59], [60], [61]]. The correlation between the two variables is stronger when the r value is higher. According to Piaw. [62], an *r* value less than 0.5 shows a 'weak’ correlation, while a range between 0.9 and 1 indicates a 'very strong’ correlation between the variables. In this study, the Pearson correlation coefficient was used to assess the degree of correspondence between the values obtained from the proposed model and the actual experimental values of compressive strength.

### 2.5 Life cycle assessment

Life cycle assessment (LCA) is a methodological framework used to evaluate the environmental performance of products and services [63]. This approach is commonly applied to assess the sustainability of buildings, construction materials, and specifically cement and concrete [64,65]. Depending on the scope, LCA may include different stages, including raw material production, manufacturing, use, and end-of-life.

The scope of this study encompasses phases A1-A3 according to EN 15804:2012+A2:2020, covering the cradle-to-gate stages [64]. The functional unit is 1 m^3^ of ready-mixed concrete, and the impact analysis methodology employed is ReCiPe Midpoint (H) V1.13 [66], including Global Warming Potential (GWP100), Terrestrial Acidification Potential (TAP100), Freshwater Eutrophication Potential (FEP), Marine Eutrophication Potential (MEP), Ozone Depletion Potential (ODPinf), and Photochemical Oxidant Formation Potential (POFP) as the impact categories. Amongst these, the Global Warming Potential, also referred to as carbon footprint, is considered the most critical indicator for climate change.

The LCA-associated calculations were conducted using OpenLCA 1.11 [67] software and the ecoinvent 3.7.1 database [68], which incorporates Ecoinvent Global (GLO) and Rest of World (RoW) environmental profiles. A ready-mix concrete plant was modeled using inventory values from Petroche et al. [69]. Since fly ash and silica fume are unavoidable by-products, no allocation of the environmental burden associated with their production was included [[70], [71], [72], [73], [74]].

## 3 Results and discussion

### 3.1 Compressive strength

**Fig. 4:**
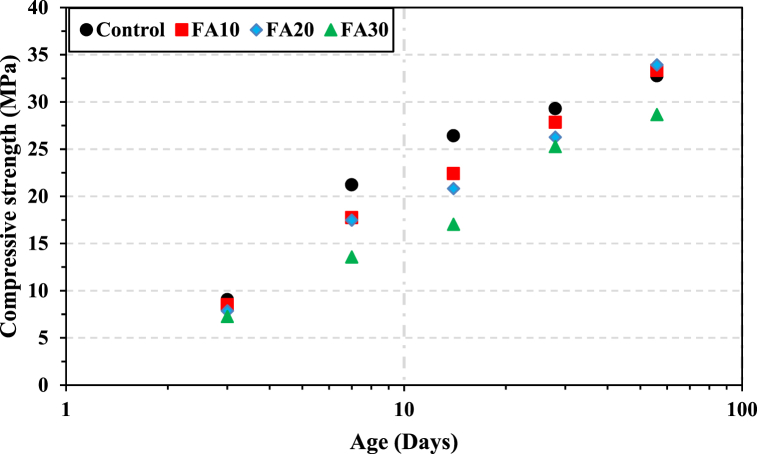
Variation in concrete compressive strength with time for different percentage contents of FA.

**Fig. 5:**
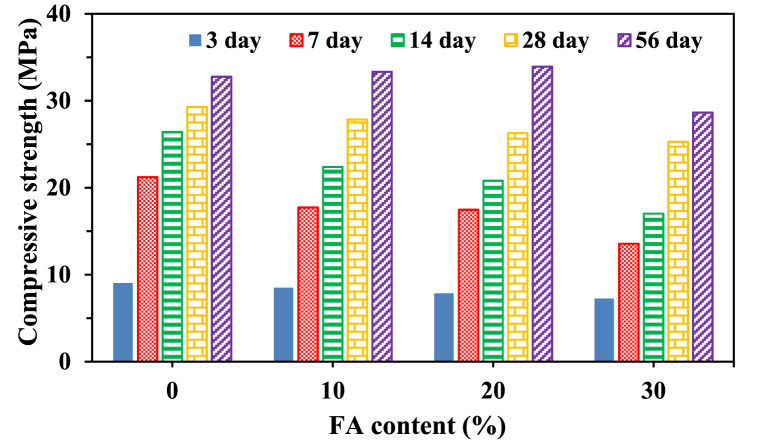
Compressive strength at different ages plotted as a function of percentage contents of FA.

The test data provides quantitative insights into the temporal evolution of the compressive strength of concrete with and without SCMs. Fig. 4 illustrates the variation of compressive strength with time for different FA content percentages (10 %, 20 %, and 30 %) along with the control. Additionally, Fig. 5 shows the variation in concrete strength at a specific age with respect to the percentage content of FA. The results depicted in Fig. 4, Fig. 5 reveal that the strength of concrete containing FA is lower than the control's strength up to a curing age of 28 days. This lower strength at the early curing age can be attributed to the low pozzolanic reactivity of fly ash during the early hydration age, primarily acting as a filler material [[75], [76], [77], [78]].

The filler effect of fly ash promotes hydration by creating nucleation sites in the microstructure, thereby forming additional calcium silicate hydrate (C–S–H) [[79], [80], [81]]. Therefore, at the later ages of 28–56 days, the compressive strength values of FA10 and FA20 gradually approach that of the control, with a strength gain of 2 % and 4 %, respectively. These results are consistent with previous findings [1,82,83]. The observed increase in strength at later ages of FA concrete is primarily attributed to the higher compactness resulting from the increased pozzolanic activity of fly ash with time [26]. However, it is essential to note that the results for concrete with a higher FA percentage (FA30) exhibited a negative effect across all ages, a trend consistent with other studies [[84], [85], [86]]. The decline in strength has been widely linked to a greater proportion of cement being replaced by FA, which reduces its cementing efficiency. This reduction may be due to the lack of Portlandite or calcium hydroxide (CH) for the pozzolanic reaction [25]. This assertion is supported by several researchers [87], who indicate that in the case of high-volume FA concrete, only part of the FA particles effectively reacts in forming C–S–H, while the remaining part remains unreactive even during the later ages of curing.

**Fig. 6:**
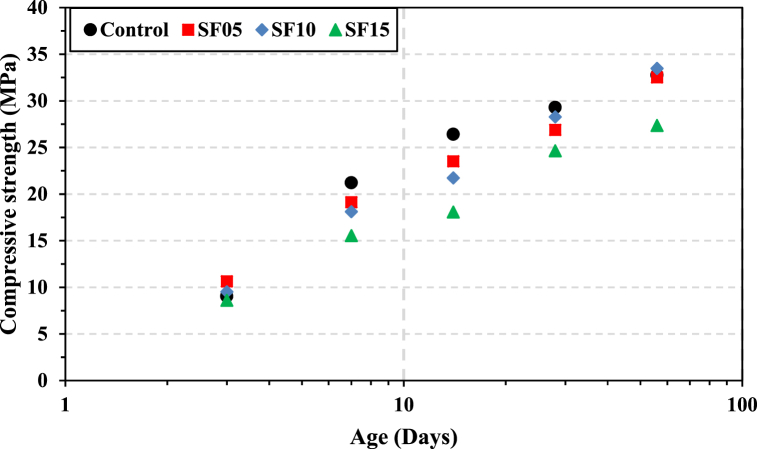
Variation in concrete compressive strength with time for different percentage contents of SF.

**Fig. 7:**
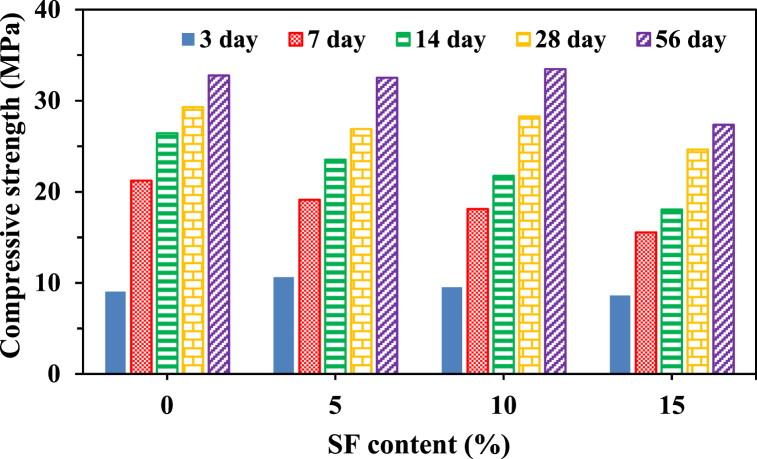
Compressive strength at different ages plotted as a function of percentage contents of SF.

The development of compressive strength with time for different percentages of SF (5 %, 10 %, and 15 %) and the control is shown in Fig. 6, while Fig. 7 displays the variation in strength concerning the percentage content of SF. At an early age of 3 days, the strength obtained was higher than that of the control concrete for SF05 and SF10, exhibiting a strength gain of 17 % and 5 %, respectively. This improvement in strength can be attributed to the densification of the matrix microstructure due to the formation of C–S–H as a result of the reaction between the microsilica from SF and the CH produced during cement hydration [88,89]. An additional factor contributing to the improved packing density of concrete, which resists greater load per unit area and increases mechanical strength, is the filling impact of ultrafine silica fume particles [[90], [91], [92]]. At 28 days, SF10 exhibited a strength comparable to the control, with a slight increase of 2 % observed at 56 days. However, the strength gain diminished for SF15 compared to the control, regardless of the curing ages. This is attributed to an excess of silica fume beyond what is required for sufficient pozzolanic reaction, where a portion of it takes part in the pozzolanic reaction while the remaining portion remains inert, thus retarding cement hydration [93]. It can be stated that the addition of SF up to 10 % resulted in increased strength, possibly due to a lower CH content resulting from the pozzolanic reaction [94]. Previous studies have shown that the best results for compressive strength were achieved when silica fume was added at partial cement replacement levels of 7 % [95] and 10 % [92,96].

The partial replacement of cement by SCMs often involves the use of certain additives, such as water-reducing agents and/or other accelerating or retarding agents, to achieve the desired concrete performance. In our study, we abstained from using any special additives or equipment to obtain the target performance, particularly in terms of compressive strength, a crucial factor in structural design computations. According to the experimental results, it can be inferred that 28-day concrete strengths with 20 % FA and 10 % SF were comparable to the target strength of 28 MPa (as mentioned earlier in Section 2.1). Interestingly, a considerable improvement in the later age strength at 56 days was noted for both cases. The experimental findings of this study suggest that the optimum dosages of cement replacement by FA and SF were approximately 15–20 % and 5–10 % of the binder, respectively.

### 3.2 Development of strength evolution model (SEM)

The strength of concrete can be described as an asymptotic evolution of the compressive strength *f*_*c*_ as a function of concrete age *t*. The following general model (Equation (1)) represents *f*_*c*_ as the response and *t* as a predictor:

(1) $$f_{c}\left(t\right)=\frac{m\;t}{n+t}$$

Where *f*_*c*_ is the compressive strength at any age (*t*) of concrete, *m* is the asymptotic value, i.e., the maximum value of *f*_*c,*_ after which the rate of change of strength is negligible. The value of *m* may be considered as 90–95% of the maximum concrete strength. While many researchers use the compressive strength value at 28 days as the reference value for assessing strength evolution [33,34,97], in this study, we used the asymptotic value of the strength, *m,* determined from the concrete specimens. Here, *n* is the concrete age when the response strength *f*_*c*_ equals half of *m,* i.e., *m*/2. Both *m* and *n* were optimized based on the method of least squares, resulting in the least sum of squared differences between the measured strength, *y,* and the corresponding calculated values *f* (*t*_*i*_, *m*, *n*), i.e., Equation (2) attains the minimum value.

(2) $$\sum_{i=1}^{n}{\left[f_{c}\left(t_{i},m,n\right)-y\left(t_{i}\right)\right]}^{2}\;\to \;\min$$

**Fig. 8:**
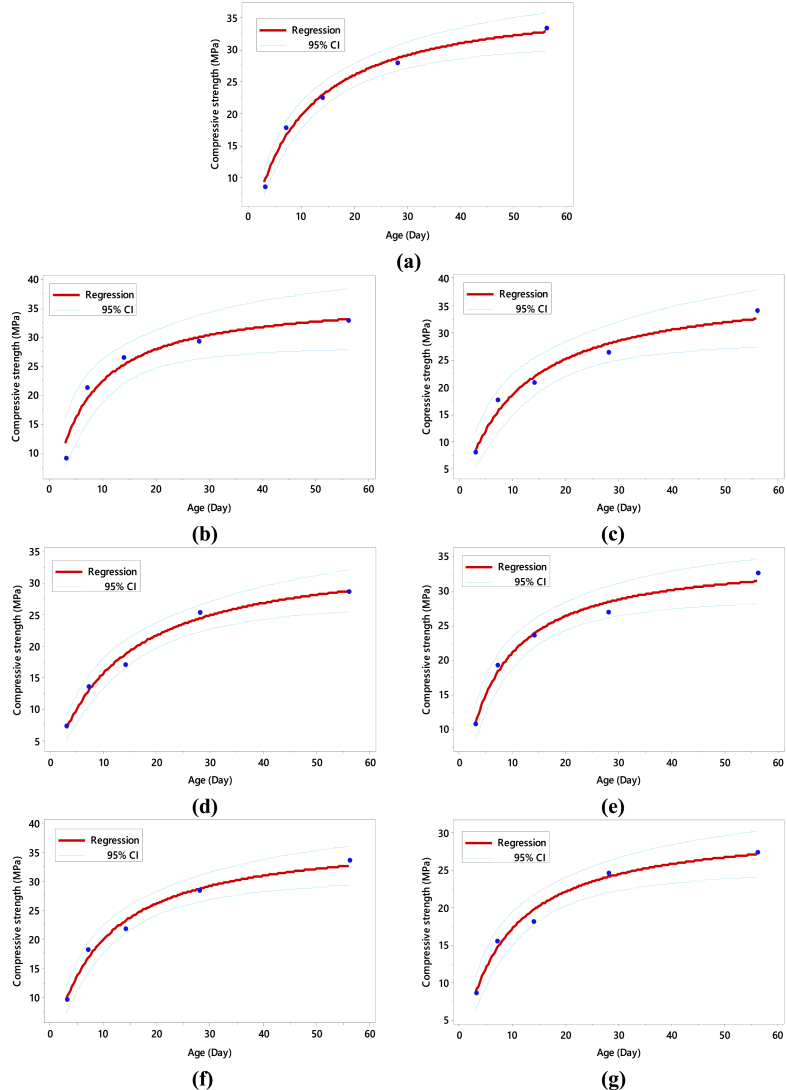
Experimental values (represented by blue dots) of compressive strength and the reproduced compressive strength (regression line) versus age of concrete: **(a)** Control; **(b)** FA10; **(c)** FA20; **(d)** FA30; **(e)** SF05; **(f)** SF10; **(g)** SF15. (For interpretation of the references to color in this figure legend, the reader is referred to the Web version of this article.)

**Fig. 9:**
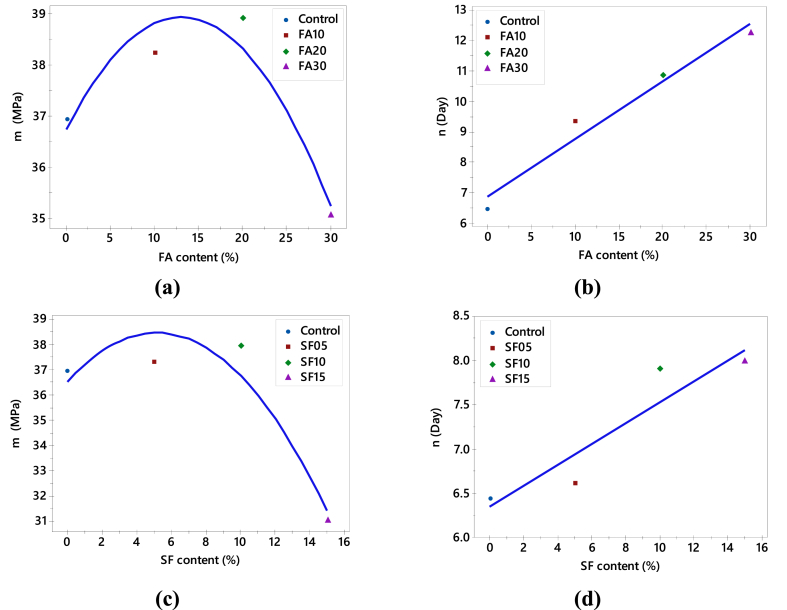
**(a) & (b)** correspond to the variation in the value of *m* and *n* to the percentage content of FA; **(c) & (d)** correspond to the variation in the value of *m* and *n* to the percentage content of SF.

A nonlinear regression analysis of Equation (1) was performed for each concrete mix to obtain the corresponding values of the parameters *m* and *n* using the 95 % confidence intervals of both the estimated and measured compressive strength values. The results of the regression analysis for all concrete types are shown in Fig. 8. The curves for control (Fig. 8a), FA (Fig. 8 b, Fig. 8 c and Fig. 8 d) and SF (Fig. 8e, Fig. 8f and Fig. 8g) illustrating the upper and lower limits of the 95 % confidence interval of the expected values for all *m* and *n* are within the range of approximately ± 5 % of the calculated values. It is also worth noting that the curves representing the upper and lower boundaries of the 95 % confidence interval are also within the range mentioned above. Interestingly, the value of *m* (refer to Fig. 9a) increases up to 20 % of the content of FA and then starts decreasing up to a 30 % replacement by FA. Conversely, for SF (depicted in Fig. 9c), *m* increases up to a 10 % replacement by SF and then displays a decreasing trend up to 15 %. This suggests that adding SCMs, such as FA and SF in this context, significantly impacts the asymptotic strength up to the optimal percentage contents of these SCMs.

As depicted in Fig. 9a, it is evident that FA concrete attains a slightly higher asymptotic value for FA10 and FA20, whereas the *m* value for FA30 is less than that of the control concrete. This aligns with previous findings, indicating that in concrete with higher FA contents, the hydration process of cement is delayed due to dilution effects resulting from FA replacing cement [25,87]. The delay in strength gain and reduction in asymptotic strength are observed with higher percentages of FA. Regarding SF concrete (see Fig. 9c), slightly different results were observed compared to FA concrete. The asymptotic values of *m* obtained for SF05 and SF10 are slightly higher than those of the control specimen, followed by a significantly lower value for SF15.

The highly reactive nature and large surface area provided by SF accelerate the pozzolanic reaction between the siliceous phase from SF and the CH resulting from cement hydration, significantly impacting concrete strength, particularly at early ages. Consequently, higher strength gain was found in concrete containing SF at early ages [88,89]. The rate of strength gain is crucial, as depicted in the evolution of the value of *n* against the percentage contents of SCMs in Fig. 9b and d. Notably, the linear increasing trend in the case of FA (see Fig. 9b) means that higher percentage contents require more time for the concrete to attain half of the maximum or asymptotic strength. A similar linear trend is observed for SF (see Fig. 9d) but not as smooth as that of FA in Fig. 9b. For SF05, the *n* value is almost identical to the control. Comparatively, it can be inferred that the rate of strength gains for concrete containing SF is faster than that of FA.

From the results in Fig. 9, the general parameters *m* and *n* of Equation (1) can be taken as functions (parabolic and linear, respectively) of the percentage contents of the SCMs. The expressions to estimate the strengths of FA- and SF-based concretes are represented as:

(3) $$f_{c,FA}=\frac{\left(36.74+33.55x-128.5x^{2}\right)\;t}{\left(6.88+18.96x\right)+t}$$

(4) $$f_{c,SF}=\frac{\left(36.55+75.13x-\;729x^{2}\right)\;t}{\left(6.34+11.82x\right)+t}$$

Where *x* is the percentage content of FA and SF within the investigated replacement levels of cement up to 30 % and 15 %, *t* represents the time in days, and *f*_*c, FA,*_ and *f*_*c, SF*_ denote the corresponding predicted strengths of FA- and SF-based concretes in MPa.

**Fig. 10:**
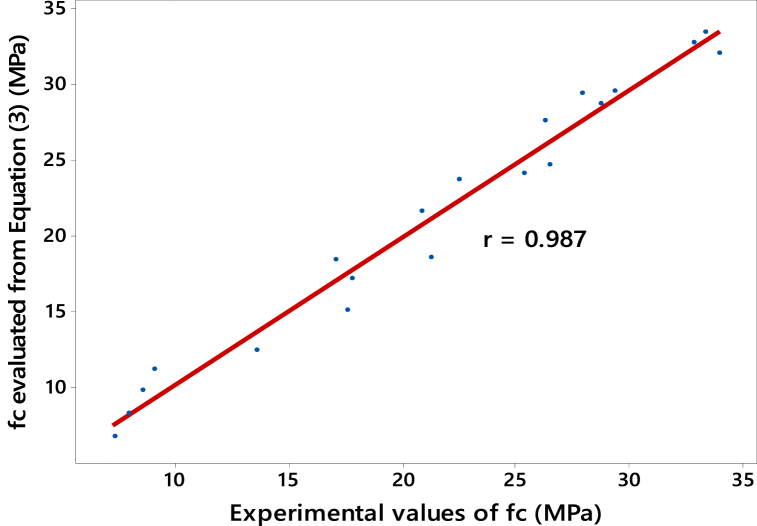
Correlation between compressive strength *f*_*c*_ evolution of FA-based concretes: Model values from Equation (3) and experimentally determined *f*_*c*_.

**Fig. 11:**
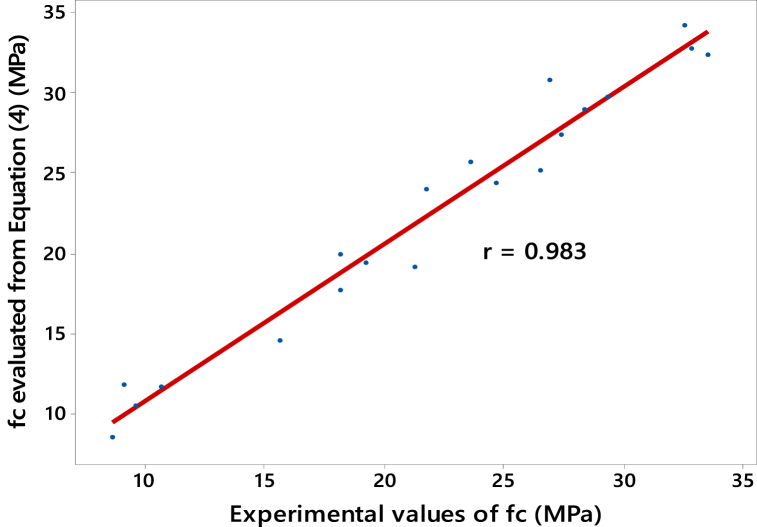
Correlation between compressive strength *f*_*c*_ evolution of SF-based concretes: Model values from Equation (4) and experimentally determined *f*_*c*_.

The comparison between the model-predicted strength values derived from Equation (1) and the experimental results is illustrated in Fig. 10, Fig. 11 for FA and SF-based concretes, respectively. Upon visual inspection of these figures, it is evident that the model values demonstrate a strong correlation with the experimental values of compressive strength. This is quantitatively supported by the higher values of the Pearson correlation coefficient, i.e., *r* = 0.987 and 0.983 (see Fig. 10, Fig. 11, respectively). Therefore, it can be established that Equation (1) effectively reproduces the compressive strength, *f*_*c*_, for each SCM used at any curing age.

### 3.3 Comparison of SEM with fib Model Code 2010

The *fib* Model Code 2010 [34,98] provides the evolution formula (Equation (5)) that establishes a relationship between early-age strength and compressive strength at 28 days, *f*_*c*_*,*_*28d*_:

(5) $$f_{c}\left(t\right)={f_{c}}_{28d}\;\exp\left[s\left(1-\sqrt{\frac{28\;days}{t}}\right)\right]$$

Where *t* represents the time in days, *s* is a dimensionless parameter linked to the rate at which the 28-day compressive strength is attained. A higher *s* value indicates a slower early-age strength evolution and vice versa. Generally, *s* depends on the 28-day strength and the cement type used in concrete production. Based on the findings of Ausweger et al. [33], it has been demonstrated that the rate of strength gain is faster compared to the rates specified in prior works by Walraven et al. [34] and Bentz [98]. For this study, a value of *s* equal to 0.38 has been considered for ordinary cement with a strength of 33 MPa at 28 days. Furthermore, applying this formula for evaluating the 56-day strength has shown good agreement between the experimental and model values.

**Fig. 12:**
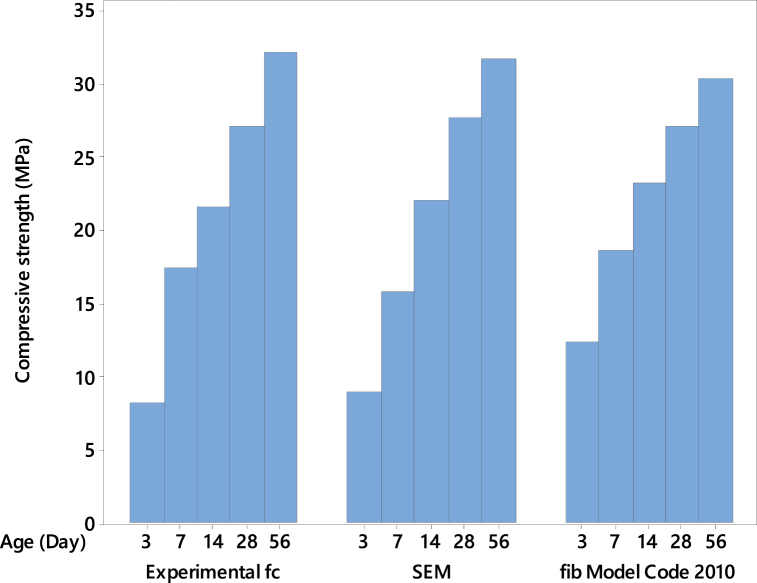
Comparison between the model and experimental values of FA-based concretes.

**Fig. 13:**
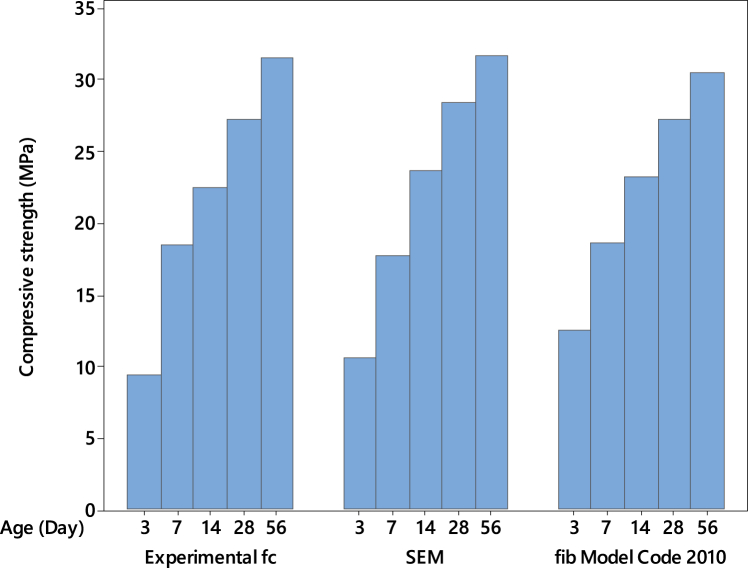
Comparison between the model and experimental values of SF-based concretes.

The *fib* Model Code 2010 (Equation (5)) was applied to the experimental data to compare the newly developed SEM. The results are shown in Fig. 12, Fig. 13 for FA- and SF-based concretes, respectively. In these figures, for comparison purposes and to denote the strength values, the mean of the compressive strength at each day was taken for the replacement levels of 0, 10, 20, and 30 % for FA-based concretes and 0, 5, 10, and 15 % for SF-based concretes. The model results and the experimental strength values in Fig. 12 are closely matched for FA-based concrete at all ages. However, at the early age of 3 days, the *SEM* has yielded a decrease of almost 30 % in strength compared to the *fib* Model Code 2010. On the other hand, when observing SF-based concrete in Fig. 13, all the model and experimental compressive strength values demonstrate good agreement across respective ages. The greatest percent change for the *SEM* compared to the *fib* Model is only 12 % at the early age of 3 days.

### 3.4 Life cycle impact assessment

**Fig. 14:**
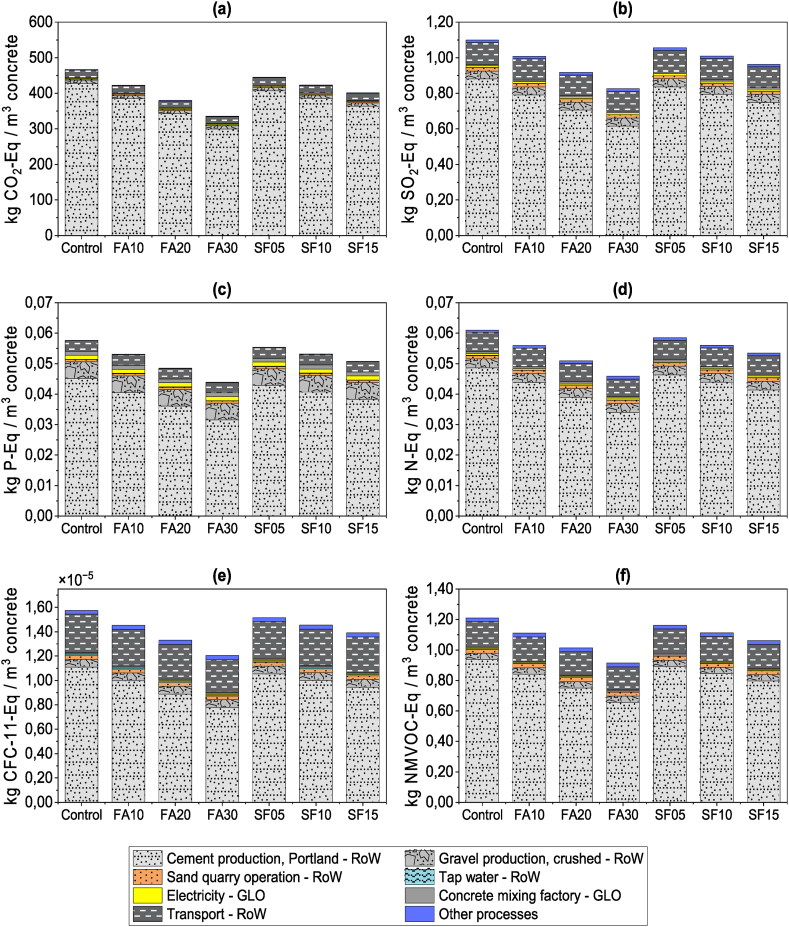
Characterization results for impact categories. The graphs show the impact results per 1 m^3^ of ready-mixed concrete (functional unit) and a contribution analysis for processes. (a) Global Warming Potential - GWP100. (b) Terrestrial Acidification Potential - TAP100. (c) Freshwater Eutrophication Potential - FEP. (d) Marine Eutrophication Potential - MEP. (e) Ozone Depletion Potential – ODPinf. (f) Photochemical Oxidant Formation Potential - POFP. GLO: Global. RoW: Rest of World.

The life cycle impact assessment (LCIA) indicator results for the control and the six mix designs included in this study are summarized in Table 3. Additionally, Fig. 14 shows the contribution results for the selected impact categories.

**Table 3:** Result of life cycle evaluation impact categories for 1 m^3^ of ready-mixed concrete.

Impact categories	Unit	Control	FA10	FA20	FA30	SF05	SF10	SF15
Global Warming Potential - GWP100	kg CO_2_-Eq	466.77	423.00	380.09	336.33	445.32	423.86	401.55
Terrestrial Acidification Potential - TAP100	kg SO_2_-Eq	1.10	1.01	0.92	0.83	1.06	1.01	0.96
Freshwater Eutrophication Potential - FEP	kg P-Eq	5.77x10^−02^	5.31 x10^−02^	4.86 x10^−02^	4.39 x10^−02^	5.55 x10^−02^	5.32 x10^−02^	5.08 x10^−02^
Marine Eutrophication Potential - MEP	kg N-Eq	0.061	0.056	0.051	0.046	0.059	0.056	0.053
Ozone Depletion Potential - ODP	kg CFC^−11^-Eq	1.57x10^−05^	1.45 x10^−05^	1.33 x10^−05^	1.21 x10^−05^	1.51 x10^−05^	1.45 x10^−05^	1.39 x10^−05^
Photochemical Oxidant Formation Potential - POFP	kg NMVOC-Eq	1.21	1.11	1.01	0.92	1.16	1.11	1.06

Substituting cement with fly ash (FA) yields environmental benefits to the concrete profile. Regarding GWP100, incorporating 10 %, 20 %, and 30 % of FA reduces carbon footprint by 9 %, 19 %, and 29 %, respectively. On average, every 1 % of cement replaced with FA represents 4.35 kg CO_2_-Eq/m^3^ of ready-mixed concrete. Using 5 %, 10 %, and 15 % of silica fume (SF) reduces the carbon footprint of concrete by 5 %, 9 %, and 14 %, respectively, based on GWP100. On average, substituting 1 % of cement with SF reduces 4.30 kg CO_2_-Eq/m^3^ of ready-mixed concrete, which is almost similar to FA. This trend can be attributed to fly ash and silica fume being unavoidable by-products that do not incur environmental burdens from their production.

Cement production has the highest contribution across all impact categories (Fig. 14). In terms of GWP100 (Fig. 14a), it constitutes an average of 90 % of this indicator for FA and SF. Compared to the control mix, reducing cement content has resulted in a 2 % decrease in its contribution. During cement production, kilns generate CO_2_, NO_X_, SO_2_, VOC, PM, and other substances through combustion and chemical reactions when calcium carbonate (CaCO_3_) transforms into calcium oxide (CaO) [2,69,99]. The substantial amount of cement required for each cubic meter of conventional ready-mix concrete (between 250 and 400 kg/m^3^) significantly contributes to these impact categories.

The second process influencing impact categories GWP100 (Fig. 14a), TAP100 (Fig. 14b), MEP (Fig. 14d), ODPinf. (Fig. 14e), POFP (Fig. 14f) is transportation. Its contribution ranges from 4.55 % to 5.51 % of the GWP100 for FA, with values from 18.54 to 20.33 kg CO_2_-Eq/m^3^ of ready-mixed concrete. For SF, its impact ranges between 4.67 and 5.95 % of the GWP100 with values between 19.89 and 20.79 kg CO_2_-Eq/m^3^ of ready-mixed concrete, as shown by a comparative analysis of different mixtures. This analysis includes the transportation of FA and SF.

Regarding transportation modeling, this study employs Ecoinvent's “market activities,” incorporating global average distances and transportation types from the producer to the consumer. It is important to note that these distances and transportation methods may differ based on the market region. Even though no environmental burdens were considered from the production of fly ash (FA) and silica fume (SF), their transportation still incurs an impact of approximately 1 % in all impact categories.

Other processes, such as the production of aggregates, water, electricity, and the construction of facilities, contribute between 4.25 % and 5.44 % to the GWP100 indicator.

Previous research that has explored the use of fly ash as a substitute for cement in concrete has utilized percentages ranging from 0 % to 40 %, calculating their carbon footprint. In a study conducted by Das et al. [100], significant reductions in the carbon footprint were observed, reaching up to 77 % when substituting 40 % of the cement with fly ash. Similarly, a 20 % substitution resulted in a 37 % reduction in the carbon footprint. In contrast, the results of this study differ, as a 20 % substitution of fly ash led to an 18.57 % reduction in the footprint. Research carried out by Cassiani et al. [101] indicates that with a 20 % substitution of fly ash, reductions of 15.03 % in the carbon footprint are obtained, which more closely resembles the results obtained in this study. Discrepancies in the results can be attributed to various factors, especially the estimated allocation for fly ash, transportation, among others.

Regarding the utilization of silica fume, Nayır et al. [102] conducted studies involving replacements of 10 % and 15 % of SF, resulting in reductions of GWP by 7.2 % and 11.72 %, respectively. Cassiani et al. [101], using a 10 % replacement of SF, achieved a reduction of 5.18 %. Delnavaz et al. [103] employed replacements ranging from 5 % to 11 %, yielding GWP reductions between 4 % and 9 %. In our study, utilizing replacements of 5 %, 10 %, and 15 %, we observed GWP reductions of 4.6 %, 9.19 %, and 13.97 %, respectively. The values obtained in this study align closely with those reported in the literature, with discrepancies attributed to factors such as the allocated estimation for silica fume, transportation, compressive strength, etc.

## 4 Conclusions

Partial replacement of cement with two commonly available supplementary cementitious materials (SCMs), fly ash and silica fume, was studied in this research to develop a new strength evolution model and investigate the benefits of using these SCMs in concrete. Based on the findings of this study, the following conclusions can be drawn.

1. The experimental results for compressive strength showed that adding fly ash (FA) in concrete resulted in a slight increase in strength at a later age (56 days) compared to the control.
2. The incorporation of silica fume (SF) improved the compressive strength both at the early and later ages of concrete.
3. An optimum value of partial cement replacement with FA was found in the range of 15–20 %, and for SF, it ranged between 5 and 15 %.
4. The proposed strength evolution model (SEM) can be used to estimate compressive strength with high precision. Moreover, it provides insight into the optimum percentages of FA and SF, the maximum possible asymptotic value of compressive strength, and opens a discussion regarding the asymptotic compressive strength and the age at which half of this strength can be achieved. These results can be beneficial for scientists and engineers working in the field of concrete and construction.
5. The model-fitted compressive strength values from the strength evolution model developed in this study were compared with the *fib* Model Code, and the results showed a close agreement.
6. The life cycle assessment (LCA) results concluded that using reduced cement contents remains the most efficient approach to enhance the environmental performance of ready-mix concrete, considering that the effects of cement production constitute roughly 90 % of all processes. The use of SCMs significantly contributes to this objective. Transporting SCMs has minimal impacts (averaging 1 %) compared to the environmental benefits of reducing overall environmental impacts.

While the regression analysis in this study produced promising results, there are still challenges due to the small number of variables considered. Therefore, it is imperative to conduct further regression-based studies using large datasets of compressive strength to validate the accuracy of this study's proposed strength evolution model (SEM). A more advanced model can incorporate a wide array of variables, including but not limited to SCMs content, water-to-cement ratio, curing age, and curing temperature. Developing such a model will serve as a reliable tool for accurately predicting the compressive strength of concrete. Moreover, it will enable the identification of key variables and their quantitative impact on compressive strength.

## Data availability

The data that support the findings of this study are available on request from the authors.

## CRediT authorship contribution statement

**Noor Yaseen**: Writing – original draft, Writing – review & editing, Visualization, Methodology, Software, Investigation, Conceptualization. **Stefany Alcivar-Bastidas**: Writing – original draft, Visualization, Resources. **Muhammad Irfan-ul-Hassan**: Writing – original draft, Visualization, Methodology, Supervision, Investigation, Writing – review & editing. **Daniel M. Petroche**: Writing – review & editing, Writing – original draft, Visualization, Resources, Investigation. **Asad Ullah Qazi**: Writing – review & editing, Conceptualization, Supervision. **Angel D. Ramirez**: Writing – review & editing, Writing – original draft, Supervision, Methodology, Conceptualization.

## Declaration of competing interest

The authors declare that they have no known competing financial interests or personal relationships that could have appeared to influence the work reported in this paper.

## Figures and Tables

**Table A:** Hydration degree as a function of curing time for a w/c ratio of 0.54 [105].

Concrete age (days)	Hydration degree (%)
3	0.44
7	0.54
14	0.63
28	0.67
56	0.74

**Fig. A:**
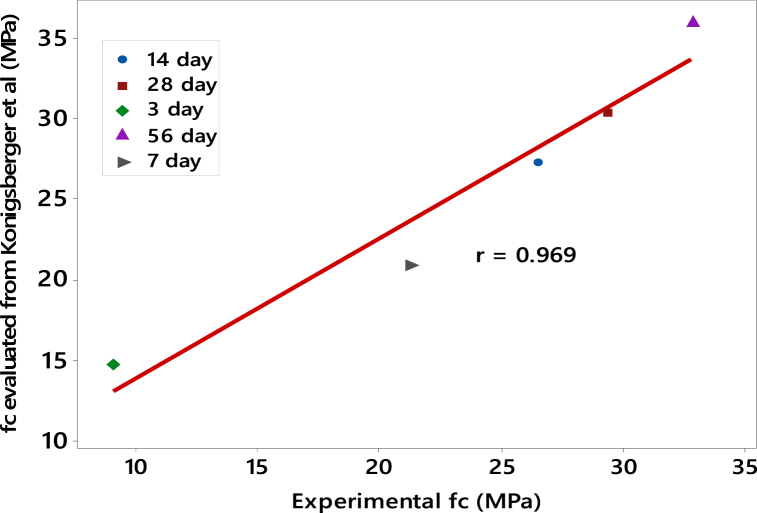
Comparison between *f*_*c*_ approximated from Königsberger et al. [58] and experimental *f*_*c*_ of control concrete.

## Appendix A Application of the multiscale model to the experimental data

To validate the experimental results of this study, the compressive strength results for the control specimens, as a representation of the experimental data, were subjected to an explicit set of formulae proposed by Königsberger et al. [58]. These formulae enable the prediction of the uniaxial compressive strength of concrete as a function of the composition of the material and maturity, characterized by the effective water-to-cement ratio, the aggregate-to-cement ratio, and the degree of hydration. To achieve this, the degrees of hydration of normal cement against various ages, considering a w/c ratio of 0.54, were taken from Cook et al. [100], as shown in Table A. In addition, the set of formulae mentioned above included the water absorption capacity of aggregates set at 0.0099 and the void-filling extent of water at 0.603, extracted from the research by Irfan ul Hassan et al. [104]. The water absorption capacity of aggregate indicates that 1 kg of quartz takes up 9.9 g of water during the mixing of the raw materials.

Moreover, it was observed that shrinkage-induced voids of the cement paste matrix suck water back from the open porosity of quartz, resulting in these voids being water-filled to a level of 60.3 % [104]. A strong alignment was observed between the values of the predicted compressive strength for the control concrete and their respective experimental results (see Figure A). Consequently, the approximated formulae based on the analytical background of concrete provided an accurate approximation of its uniaxial compressive strength. Consequently, the experimental results of this study were appropriately validated using a multiscale model, and hence, the experimental data used for regression and correlation analyses were adequate.
